# An extreme value analysis of daily new cases of COVID-19 for sixteen countries in west Africa

**DOI:** 10.1038/s41598-023-37722-9

**Published:** 2023-07-04

**Authors:** Saralees Nadarajah, Oluwadare O. Ojo

**Affiliations:** 1 Department of Mathematics, University of Manchester, Manchester, M13 9PL UK; 2 Department of Statistics, Federal University of Technology, Akure, Nigeria

**Keywords:** Diseases, Medical research, Mathematics and computing

## Abstract

We provide an extreme value analysis of daily new cases of COVID-19. We use data from Benin, Burkina Faso, Cabo Verde, Cote d’Ivoire, Gambia, Ghana, Guinea, Guinea-Bissau, Liberia, Mali, Mauritania, Niger, Nigeria, Senegal, Sierra Leone and Togo, covering a period of 37 months. Extreme values were defined as monthly maximums of daily new cases. The generalized extreme value distribution was fitted to them with two of its three parameters allowed to vary linearly or quadratically with respect to month number. Ten of the sixteen countries were found to exhibit significant downward trends in monthly maximums. The adequacy of fits was assessed by probability plots and the Kolmogorov-Smirnov test. The fitted models were used to derive quantiles of the monthly maximum of new cases as well as their limits when the month number is taken to infinity.

## Introduction

There have been many papers published on daily new cases and daily new deaths of the COVID-19 pandemic. But often the interest is with the extreme values related to these variables; for example, monthly maximums of daily new cases or monthly maximums of daily new deaths. We are aware of only a few papers on extreme values related to the COVID-19 pandemic. Omari et al.^1^ forecasted value-at-risk of financial markets under the global pandemic of COVID-19 using conditional extreme value theory. Hernandy et al.^2^ estimated extreme values of COVID-19 cases using an exponential smoothing Holt-Winters based-approach. Ibrahim et al.^3^ predicted COVID-19 spread using compartmental model and extreme value theory with application to Egypt and Iraq. Khan et al.^4^ investigated the extreme tail behavior of NIFTY 50 index during the COVID-19 pandemic. Eliwa and El-Morshedy^5^ used a COVID-19 data to illustrate the fit of a distribution. Enriquez et al.^6^ fitted the Gumbel distribution to daily new cases from Argentina, Brazil, China, Colombia, France, Germany, India, Indonesia, Iran, Italy, Mexico, Poland, Russia, Spain, the United Kingdom, and the United States. Winkler et al.^7^ investigated shifts in time use behavior during the COVID-19 pandemic.

Except for Enriquez et al.^6^, none of these papers appear to provide an extreme value analysis of daily new cases or daily new deaths. Enriquez et al.^6^ used the Gumbel distribution, a particular extreme value distribution. Besides, they accounted for no trends in the data. The aim of this paper is to provide an analysis for daily new cases using a general extreme value distribution and accounting for trends in data. We use data on daily new cases from sixteen countries in west Africa. We take extreme values as the monthly maximums of daily new cases.

Suppose $Y_1, \ldots , Y_m$ are reported new cases over *m* days. The maximum of the new cases is $\max \left( Y_1, \ldots , Y_m \right) = X$ say. There is a probability that *X* can be equal to zero. Let $\Pr (X = 0) = p$. If $X > 0$, then under suitable conditions, a normalized version of the distribution of *X* can be shown to converge to one of Gumbel, Fréchet or Weibull distributions^8,9^ specified by the cumulative distribution functions

$$\begin{aligned}{} & {} \displaystyle {\text{ Gumbel:}} \qquad \exp \left[ -\exp \left( -\frac{x - \mu }{\sigma } \right) \right] ,\\{} & {} \displaystyle \hbox {Fr}\acute{\textrm{e}}\hbox {chet: } \qquad \exp \left[ -\left( \frac{x - \mu }{\sigma } \right) ^{-\alpha } \right] \end{aligned}$$

and

$$\begin{aligned} \displaystyle \text{ Weibull: } \qquad \exp \left[ -\left( -\frac{x - \mu }{\sigma } \right) ^{\alpha } \right] , \end{aligned}$$

respectively, as $m \rightarrow \infty$ for $-\infty< \mu < \infty$, $\sigma > 0$ and $\alpha > 0$. Jenkinson^10^ showed that the Gumbel, Fréchet and Weibull distributions can be combined into one distribution referred to the generalized extreme value distribution specified by the cumulative distribution function

1 $$\begin{aligned} \displaystyle \exp \left[ -\left( 1 + \xi \frac{x - \mu }{\sigma } \right) ^{-\frac{1}{\xi }} \right] \end{aligned}$$

for $\mu - \frac{\sigma }{\xi } \le x < \infty$ if $\xi > 0$, $-\infty< x < \infty$ if $\xi = 0$ and $-\infty < x \le \mu - \frac{\sigma }{\xi }$ if $\xi < 0$, where $-\infty< \mu < \infty$ denotes a location parameter, $\sigma > 0$ denotes a scale parameter and $-\infty< \xi < \infty$ denotes a shape parameter. Note that if $\xi > 0$ then *X* has a heavy tail bounded below by $\mu - \frac{\sigma }{\xi }$. If $\xi < 0$ then *X* has a short tail bounded above by $\mu - \frac{\sigma }{\xi }$.

If *m* is large enough then the distribution of *X*, the monthly maximum of new cases, conditioned on $X > 0$, can be approximated by (1). We refer to the distribution of $X \mid X > 0$ being approximated by (1) as the generalized extreme value model. The properties of the generalized extreme value model including estimation methods, prediction methods, simulation methods and extensions have been studied by many authors. We refer the readers to Leadbetter et al.^11^, Galambos^12^, Resnick^13^, Embrechts et al.^14^, Kotz and Nadarajah^15^, Coles^16^, Beirlant et al.^17^, Gumbel^18^, Castillo et al.^19^, Malevergne and Sornette^20^, de Haan and Ferreira^21^, Reiss and Thomas^22^, Falk et al.^23^, Novak^24^, Ahsanullah^25^ and references therein for details.

In this paper, we fit the generalized extreme value model to the data with its location and scale parameters allowed to vary linearly or quadratically with respect to month number. The fitted models were used to infer quantiles of the monthly maximum of new cases as well as their limits when the month number is taken to infinity.

The contents of the paper are organized as follows. Section “Data” describes the data. The generalized extreme value model accommodating various trends is described in “Section Models”. The results of fitting the model and their discussion are provided in “Section Results and discussion”. Finally, some conclusions are noted in “Section Conclusions”.

## Data

**Figure 1:**
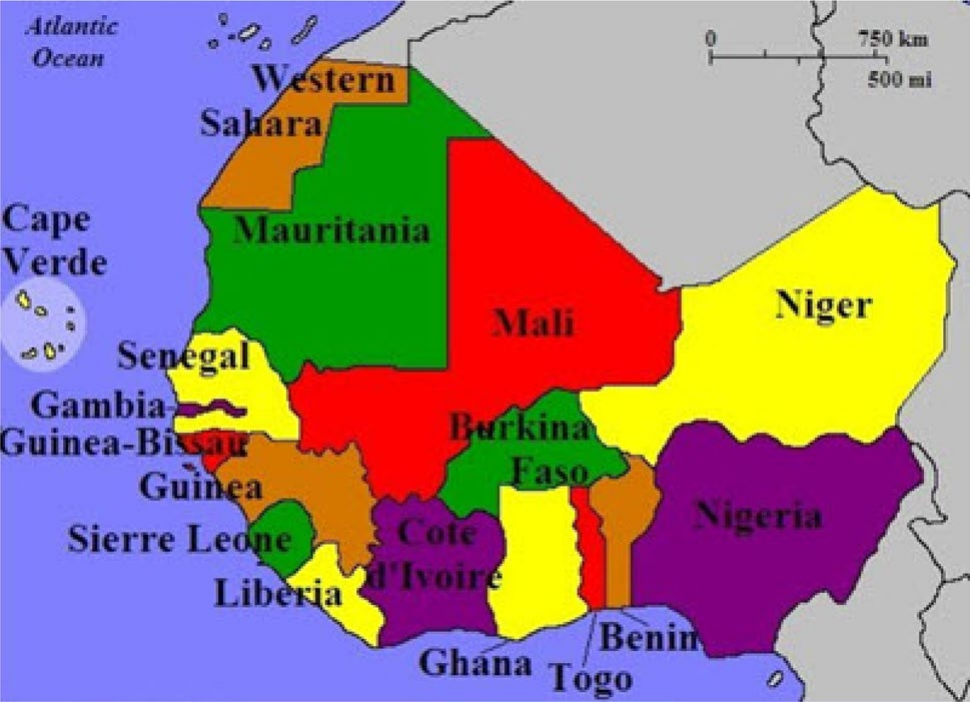
The countries in west Africa.

The data are daily new cases in Benin, Burkina Faso, Cabo Verde, Cote d’Ivoire, Gambia, Ghana, Guinea, Guinea-Bissau, Liberia, Mali, Mauritania, Niger, Nigeria, Senegal, Sierra Leone and Togo (see Fig. 1) from the 3rd of January 2020 to the 16th of February 2023. The data were downloaded from https://ourworldindata.org/covid-cases The data of interest are monthly maximums of daily new cases. If $z_3, z_4, \ldots , z_{366}, z_{367}, z_{368}, \ldots , z_{731}, z_{732}, z_{733}, \ldots , z_{1096}, z_{1097}, z_{1098}, \ldots , z_{1127}$ enumerate the daily values from the 3rd of January 2020 to the 31st of January 2023 then the monthly maximums are $\max \left( z_3, \ldots , z_{31} \right)$, $\max \left( z_{32}, \ldots , z_{61} \right)$, $\ldots$, $\max \left( z_{336}, \ldots , z_{366} \right)$, $\max \left( z_{367}, \ldots , z_{397} \right)$, $\max \left( z_{398}, \ldots , z_{425} \right)$, $\ldots$, $\max \left( z_{701}, \ldots , z_{731} \right)$, $\max \left( z_{732}, \ldots , z_{762} \right)$, $\max \left( z_{763}, \ldots , z_{790} \right)$, $\ldots$, $\max \left( z_{1066}, \ldots , z_{1096} \right)$ and $\max \left( z_{1098}, \ldots , z_{1127} \right)$. The corresponding month numbers are $1, 2, \ldots , 37$. Some summary statistics of this data are shown in Table 1.

**Table 1:** Some summary statistics of the maximum of daily new cases.

Country	Minimum	First quartile	Median	Mean	Third quartile	Maximum	Variance	Skewness	Kurtosis
Benin	0	31	86	264.270	151.000	3440.000	1860954.985	1.770	4.165
Burkina Faso	0	14	40	120.811	136.000	1005.000	151270.514	1.707	4.052
Cabo Verde	0	17	74	153.541	149.000	1469.000	326265.097	1.738	4.110
Cote d’Ivoire	0	49	115	291.514	363.000	2858.000	1229549.439	1.725	4.085
Gambia	0	5	19	59.000	75.000	376.000	20668.400	1.620	3.889
Ghana	0	133	372	575.243	866.000	2702.000	987342.050	1.420	3.534
Guinea	0	47	100	122.865	153.000	438.000	23808.611	1.289	3.403
Guinea-Bissau	0	12	28	53.919	69.000	305.000	13025.979	1.591	3.845
Liberia	0	4	11	30.405	26.000	219.000	7128.521	1.709	4.056
Mali	0	13	42	95.189	87.000	1217.000	229441.523	1.763	4.154
Mauritania	0	19	65	196.000	179.000	2545.000	1009613.067	1.763	4.153
Niger	0	9	16	34.541	33.000	301.000	13481.538	1.735	4.104
Nigeria	0	120	296	593.676	790.000	4035.000	2337177.020	1.644	3.938
Senegal	0	17	80	192.135	181.000	1722.000	448148.100	1.732	4.097
Sierra Leone	0	2	10	28.351	22.000	160.000	3750.356	1.660	3.966
Togo	0	14	39	95.270	61.000	747.000	84022.924	1.735	4.101

All the countries have months with no new cases. The first quartile is smallest for Sierra Leone and largest for Ghana. The median is smallest for Sierra Leone and largest for Ghana. The mean is smallest for Sierra Leone and largest for Nigeria. The third quartile is smallest for Sierra Leone and largest for Ghana. The highest of the new cases reported in a month is smallest for Sierra Leone and largest for Nigeria. The variance is smallest for Sierra Leone and largest for Nigeria. The skewness is always positive. It is smallest for Guinea and largest for Benin. All kurtosis values are greater than 3, meaning that the data are heavy tailed. The data are least heavy tailed for Guinea and most heavy tailed for Benin.

**Figure 2:**
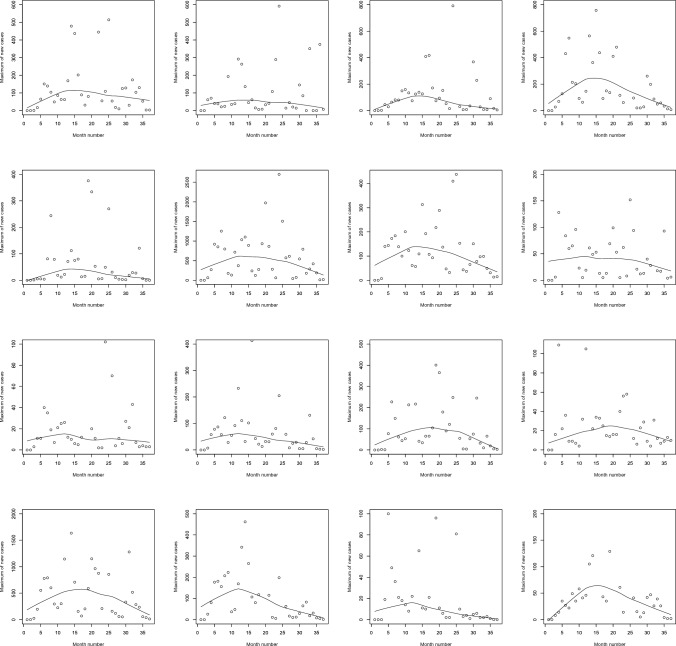
Scatter plots of the monthly maximums of daily new cases with lowess smoothing (first row, first column: Benin; first row, second column: Burkina Faso; first row, third column: Cabo Verde; first row, fourth column: Cote d’Ivoire; second row, first column: Gambia; second row, second column: Ghana; second row, third column: Guinea; second row, fourth column: Guinea-Bissau; third row, first column: Liberia; third row, second column: Mali; third row, third column: Mauritania; third row, fourth column: Niger; fourth row, first column: Nigeria; fourth row, second column: Senegal; fourth row, third column: Sierra Leone; fourth row, fourth column: Togo). The month number is counted from January 2020.

Figure 2 shows scatter plots of the data on monthly maximums for the sixteen countries. Also shown are lowess^26,27^ smoothed versions of the scatter plots. Most countries appear to exhibit significant trends with respect to month number. The trends appear either linear or quadratic.

“Section Models” assumes that the monthly maximums of new cases follow the generalized extreme value model. Estimation of the generalized extreme value model by the method of maximum likelihood requires that the data are independent (because likelihood function is defined as the product of probability density functions). We tested for independence using runs test. The corresponding *p*-values were 0.099, 0.078, 0.087, 0.067, 0.084, 0.077, 0.087, 0.077, 0.088, 0.061, 0.078, 0.065, 0.069, 0.093, 0.057 and 0.092.

## Models

**Figure 3:**
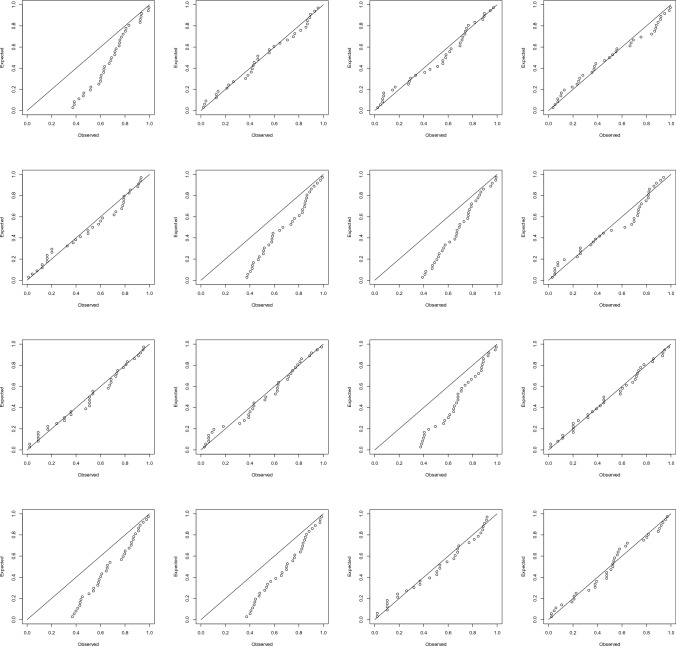
Probability plots for the fits of the generalized extreme value model (first row, first column: Benin; first row, second column: Burkina Faso; first row, third column: Cabo Verde; first row, fourth column: Cote d’Ivoire; second row, first column: Gambia; second row, second column: Ghana; second row, third column: Guinea; second row, fourth column: Guinea-Bissau; third row, first column: Liberia; third row, second column: Mali; third row, third column: Mauritania; third row, fourth column: Niger; fourth row, first column: Nigeria; fourth row, second column: Senegal; fourth row, third column: Sierra Leone; fourth row, fourth column: Togo).

Let *X* denote a random variable representing the monthly maximum of new cases. The fit of the generalized extreme value model to the data on *X*, conditioned on $X > 0$, by the method of maximum likelihood (see Coles^16^ for details) yielded the probability plots shown in Fig. 3. We see that the fits of the generalized extreme value model are generally poor.

To produce a better fit, we now account for the trends in the data noted in Section “Data”. We fitted the following models (the first referred to as the constant model is the same as the generalized extreme value model).

1. the constant model given by
  2 $$\begin{aligned}{} & {} \displaystyle \mu (x) \equiv \exp \left( a_1 \right) , \nonumber \\{} & {} \displaystyle \sigma (x) \equiv \exp \left( a_2 \right) , \nonumber \\{} & {} \displaystyle \xi (x) \equiv a_3; \end{aligned}$$
2. the linear location model given by
  3 $$\begin{aligned}{} & {} \displaystyle \mu (x) = \exp \left[ a_1 + b_1 \cdot \text{ month } \text{ no } (x) \right] , \nonumber \\{} & {} \displaystyle \sigma (x) \equiv \exp \left( a_2 \right) , \nonumber \\{} & {} \displaystyle \xi (x) \equiv a_3; \end{aligned}$$
3. the quadratic location model given by
  4 $$\begin{aligned}{} & {} \displaystyle \mu (x) = \exp \left[ a_1 + b_1 \cdot \text{ month } \text{ no } (x) + c_1 \cdot \text{ month } \text{ no } (x)^2 \right] , \nonumber \\{} & {} \displaystyle \sigma (x) \equiv \exp \left( a_2 \right) , \nonumber \\{} & {} \displaystyle \xi (x) \equiv a_3; \end{aligned}$$
4. the linear scale model given by
  5 $$\begin{aligned}{} & {} \displaystyle \mu (x) \equiv \exp \left( a_1 \right) , \nonumber \\{} & {} \displaystyle \sigma (x) = \exp \left[ a_2 + b_2 \cdot \text{ month } \text{ no } \text{(x) } \right] , \nonumber \\{} & {} \displaystyle \xi (x) \equiv a_3; \end{aligned}$$
5. the quadratic scale model given by
  6 $$\begin{aligned}{} & {} \displaystyle \mu (x) \equiv \exp \left( a_1 \right) , \nonumber \\{} & {} \displaystyle \sigma (x) = \exp \left[ a_2 + b_2 \cdot \text{ month } \text{ no } (x) + c_2 \cdot \text{ month } \text{ no } (x)^2 \right] , \nonumber \\{} & {} \displaystyle \xi (x) \equiv a_3; \end{aligned}$$
6. the linear location and linear scale model given by
  7 $$\begin{aligned}{} & {} \displaystyle \mu (x) = \exp \left[ a_1 + b_1 \cdot \text{ month } \text{ no } (x) \right] , \nonumber \\{} & {} \displaystyle \sigma (x) = \exp \left[ a_2 + b_2 \cdot \text{ month } \text{ no } (x) \right] , \nonumber \\{} & {} \displaystyle \xi (x) \equiv a_3; \end{aligned}$$
7. the quadratic location and linear scale model given by
  8 $$\begin{aligned}{} & {} \displaystyle \mu (x) = \exp \left[ a_1 + b_1 \cdot \text{ month } \text{ no } (x) + c_1 \cdot \text{ month } \text{ no } (x)^2 \right] , \nonumber \\{} & {} \displaystyle \sigma (x) = \exp \left[ a_2 + b_2 \cdot \text{ month } \text{ no } (x) \right] , \nonumber \\{} & {} \displaystyle \xi (x) \equiv a_3; \end{aligned}$$
8. the linear location and quadratic scale model given by
  9 $$\begin{aligned}{} & {} \displaystyle \mu (x) = \exp \left[ a_1 + b_1 \cdot \text{ month } \text{ no } (x) \right] , \nonumber \\{} & {} \displaystyle \sigma (x) = \exp \left[ a_2 + b_2 \cdot \text{ month } \text{ no } (x) + c_2 \cdot \text{ month } \text{ no } (x)^2 \right] , \nonumber \\{} & {} \displaystyle \xi (x) \equiv a_3; \end{aligned}$$
9. the quadratic location and quadratic scale model given by
  10 $$\begin{aligned}{} & {} \displaystyle \mu (x) = \exp \left[ a_1 + b_1 \cdot \text{ month } \text{ no } (x) + c_1 \cdot \text{ month } \text{ no } (x)^2 \right] , \nonumber \\{} & {} \displaystyle \sigma (x) = \exp \left[ a_2 + b_2 \cdot \text{ month } \text{ no } (x) + c_2 \cdot \text{ month } \text{ no } (x)^2 \right] , \nonumber \\{} & {} \displaystyle \xi (x) \equiv a_3, \end{aligned}$$

where *x* denotes the data (monthly maximum of new cases) and month no (*x*) taking values $1, 2, \ldots , 37$ denotes the month number corresponding to *x*. The parameters $b_1$ and $b_2$ correspond to linear trends with respect to month number. The parameters $c_1$ and $c_2$ correspond to quadratic trends with respect to month number.

The models given by (2) to (10) were fitted by the method of maximum likelihood by maximizing

$$\begin{aligned} \displaystyle L \left( {\varvec{\mu }}, {\varvec{\sigma }}, a_3 \right) = \prod _{i = 1}^n \left\{ \frac{1}{\sigma \left( x_i \right) } \left( 1 + a_3 \frac{x_i - \mu \left( x_i \right) }{\sigma \left( x_i \right) } \right) ^{-1 - \frac{1}{a_3}} \exp \left[ -\left( 1 + a_3 \frac{x_i - \mu \left( x_i \right) }{\sigma \left( x_i \right) } \right) ^{-\frac{1}{a_3}} \right] \right\} , \end{aligned}$$

where $\left\{ x_i: x_i > 0 \right\}$ are the data, *n* is the number of $x_i$s strictly greater than zero, $\mu \left( x_i \right)$ is given by (2)–(10), $\sigma \left( x_i \right)$ is also given by (2)–(10), ${\varvec{\mu }} = \left( a_1, b_1, c_1 \right)$ and ${\varvec{\sigma }} = \left( a_2, b_2, c_2 \right)$. The maximization was performed by using the optim function in the R software (R Development Core Team,^28^). Let $\widehat{\varvec{\mu }} = \left( \widehat{a_1}, \widehat{b_1}, \widehat{c_1} \right)$ and $\widehat{\varvec{\sigma }} = \left( \widehat{a_2}, \widehat{b_2}, \widehat{c_2} \right)$ denote that maximum likelihood estimates of $\widehat{\varvec{\mu }}$ and $\widehat{\varvec{\sigma }}$, respectively.

Standard errors/confidence intervals associated with parameters can be obtained by assuming asymptotic normality of maximum likelihood estimates and inverting the observed information matrix. But this approach supposes: (i) normality of maximum likelihood estimates; (ii) the sample size is infinity; (iii) the parameter estimates are fixed and non-random. None of these assumptions may hold in reality. A realistic approach is to use the following simulation scheme to obtain standard errors/confidence intervals^29^:

(i) simulate 10,000 samples from the fitted model each of the same size of the data;
(ii) refit the model for each of the 10,000 samples;
(iii) compute the empirical distribution out of the 10,000 estimates of the parameter of interest;
(iv) use the empirical distribution to compute the standard error/confidence interval for the parameter.

The parameters of interest could be the parameters in (2)–(10) or probabilities of interest.

## Results and discussion

We used the method in “Section Models” to model the data on monthly maximum of new cases for each of the sixteen countries. We started with constant model and then added one parameter at a time to fit the linear location, quadratic location, linear scale, quadratic scale, linear location and linear scale, quadratic location and linear scale, linear location and quadratic scale and the quadratic location and quadratic scale models. We also started with the quadratic location and quadratic scale model and subtracted one parameter at a time to fit the linear location and quadratic scale, quadratic location and linear scale, linear location and linear scale, quadratic scale, linear scale, quadratic location, linear location and the constant models. Both approaches led to the same model. The significance or non-significance of parameters (to be added or subtracted) was determined by the likelihood ratio test by comparing likelihood values^30^. We also used the Akaike information criterion due to Akaike^31^ and the Bayesian information criterion due to Schwarz^32^ to check significance or non-significance.

The values of log-likelihood, Akaike information criterion and Bayesian information criterion for (2) to (10) for Burkina Faso, one of the sixteen countries, are given in Table 2. We see that the constant model is the best fitting model. Table 3 gives the parameter estimates and standard errors of the best fitting models for all the sixteen countries. The standard errors were obtained by the bootstrap procedure outlined in “Section Models”. We see that all of the standard errors are less than the parameter estimates in magnitude.

**Table 2:** Fitted models and values of $-\log L$, AIC, BIC for Burkina Faso.

Model	$-\log L$	AIC	BIC
Constant	183.5051	373.0102	377.843
Linear location	183.5045	375.009	381.4527
Quadratic location	183.5045	377.009	385.0636
Linear scale	183.5045	375.0089	381.4526
Quadratic scale	183.5045	377.0089	385.0635
Linear location and linear scale	183.5045	377.0089	385.0635
Quadratic location and linear scale	183.5045	379.0089	388.6744
Linear location and quadratic scale	183.5045	379.0089	388.6744
Quadratic location and quadratic scale	183.5045	381.0089	392.2854

The shape parameter estimates in Table 3 are positive for all of the countries. When the constant model was fitted and $H_0: \xi = 0$ was tested, the *p*-values given in Table 4 suggest that the estimates of $\xi$ are significantly positive. When $\xi > 0$, the distribution of the monthly maximum of new cases is heavy tailed and unbounded from above. The heaviest of the tails is for Guinea, followed by Ghana, Niger, Nigeria, Mali, Benin, Togo, Cote d’Ivoire, Cabo Verde, Guinea-Bissau, Mauritania, Burkina Faso, Liberia, Senegal, Gambia and Sierra Leone.

**Table 3:** Parameter estimates and standard errors (obtained by simulation) of the best fitting models.

Country	Best fitting model	Estimates (ses)
Benin	Quadratic location	$\widehat{p} = 0.000 (0.000)$,
		$\widehat{a_1} = 3.488 (1.932)$, $\widehat{a_2} = 4.040 (1.021)$, $\widehat{a_3} = 0.970 (0.201)$,
		$\widehat{b_1} = 9.586 (2.034)$, $\widehat{c_1} = -28.463 (5.023)$
Burkina Faso	Constant	$\widehat{p} = 0.000 (0.000)$,
		$\widehat{a_1} = 3.504 (0.243)$, $\widehat{a_2} = 3.638 (0.286)$, $\widehat{a_3} = 1.072 (0.281)$
Cabo Verde	Quadratic location	$\widehat{p} = 0.000 (0.000)$,
		$\widehat{a_1} = 3.488 (0.923)$, $\widehat{a_2} = 4.040 (1.193)$, $\widehat{a_3} = 0.970 (0.322)$,
		$\widehat{b_1} = 9.586 (2.401)$, $\widehat{c_1} = -28.463 (2.033)$
Cote d’Ivoire	Quadratic location	$\widehat{p} = 0.000 (0.000)$,
		$\widehat{a_1} = 2.537 (0.512)$, $\widehat{a_2} = 3.580 (1.001)$, $\widehat{a_3} = 0.761 (0.192)$,
		$\widehat{b_1} = 30.733 (2.391)$, $\widehat{c_1} = -109.579 (9.391)$
Gambia	Constant	$\widehat{p} = 0.000 (0.000)$,
		$\widehat{a_1} = 2.455 (0.280)$, $\widehat{a_2} = 2.777 (0.316)$, $\widehat{a_3} = 1.294 (0.281)$
Ghana	Linear location	$\widehat{p} = 0.018 (0.002)$,
		$\widehat{a_1} = 5.820 (1.132)$, $\widehat{a_2} = 5.630 (1.142)$, $\widehat{a_3} = 0.523 (0.049)$,
		$$\widehat{b_1} = -1.053 (0.291)$$
Guinea	Quadratic location	$\widehat{p} = 0.138 (0.028)$,
		$\widehat{a_1} = 4.033 (0.701)$, $\widehat{a_2} = 4.027 (0.739)$, $\widehat{a_3} = 0.241 (0.038)$,
		$\widehat{b_1} = 8.011 (2.947)$, $\widehat{c_1} = -25.389 (4.411)$
Guinea-Bissau	Constant	$\widehat{p} = 0.000 (0.000)$,
		$\widehat{a_1} = 2.896 (0.259)$, $\widehat{a_2} = 2.978 (0.283)$, $\widehat{a_3} = 0.985 (0.359)$
Liberia	Constant	$\widehat{a_1} = 1.972 (0.218)$, $\widehat{a_2} = 2.029 (0.289)$, $\widehat{a_3} = 1.162 (0.288)$
Mali	Quadratic location and linear scale	$\widehat{p} = 0.000 (0.000)$,
		$\widehat{a_1} = 4.479 (0.711)$, $\widehat{a_2} = 5.117 (0.481)$, $\widehat{a_3} = 1.511 (0.086)$,
		$\widehat{b_1} = -4.571 (1.091)$, $\widehat{c_1} = -11.132 (1.701)$,
		$$\widehat{b_2} = -11.089 (1.832)$$
Mauritania	Quadratic location	$\widehat{p} = 0.000 (0.000)$,
		$\widehat{a_1} = 2.797 (0.552)$, $\widehat{a_2} = 3.754 (0.039)$, $\widehat{a_3} = 1.014 (0.024)$,
		$\widehat{b_1} = 16.863 (2.011)$, $\widehat{c_1} = -51.829 (1.011)$
Niger	Constant	$\widehat{p} = 0.000 (0.000)$,
		$\widehat{a_1} = 2.586 (0.160)$, $\widehat{a_2} = 2.380 (0.223)$, $\widehat{a_3} = 0.759 (0.209)$
Nigeria	Quadratic location	$\widehat{p} = 0.000 (0.000)$,
		$\widehat{a_1} = 4.805 (0.633)$, $\widehat{a_2} = 5.316 (0.710)$, $\widehat{a_3} = 0.953 (0.281)$,
		$\widehat{b_1} = 6.521 (1.507)$, $\widehat{c_1} = -17.319 (3.012)$
Senegal	Quadratic location and quadratic scale	$\widehat{p} = 0.000 (0.000)$,
		$\widehat{a_1} = 2.989 (0.265)$, $\widehat{a_2} = 1.906 (0.201)$, $\widehat{a_3} = 0.999 (0.122)$,
		$\widehat{b_1} = 22.016 (2.033)$, $\widehat{c_1} = -73.285 (9.045)$,
		$\widehat{b_2} = 32.617 (1.044)$, $\widehat{c_2} = -93.645 (4.861)$,
Sierra Leone	Linear location and linear scale	$\widehat{p} = 0.000 (0.000)$,
		$\widehat{a_1} = 3.895 (0.152)$, $\widehat{a_2} = 3.500 (0.131)$, $\widehat{a_3} = 1.141 (0.059)$,
		$\widehat{b_1} = -10.269 (1.912)$, $\widehat{b_2} = -9.025 (1.037)$
Togo	Constant	$\widehat{p} = 0.000 (0.000)$,
		$\widehat{a_1} = 3.218 (0.238)$, $\widehat{a_2} = 3.388 (0.244)$, $\widehat{a_3} = 0.895 (0.239)$

**Table 4:** *p*-values of the test that $\xi = 0$.

Country	Estimate and se of $\xi$	*p*-value
Benin	0.856 (0.233)	0.000
Burkina Faso	1.070 (0.281)	0.000
Cabo Verde	0.960 (0.313)	0.000
Cote d’Ivoire	0.936 (0.252)	0.000
Gambia	1.295 (0.281)	0.000
Ghana	0.487 (0.260)	0.018
Guinea	0.223 (0.075)	0.138
Guinea-Bissau	0.983 (0.359)	0.000
Liberia	1.161 (0.288)	0.000
Mali	0.820 (0.275)	0.000
Mauritania	1.020 (0.329)	0.000
Niger	0.758 (0.209)	0.000
Nigeria	0.771 (0.267)	0.000
Senegal	1.171 (0.289)	0.000
Sierra Leone	1.342 (0.324)	0.000
Togo	0.896 (0.239)	0.000

According to Table 3, the constant model gave the best fit for Burkina Faso, Gambia, Guinea-Bissau, Liberia, Niger and Togo. The linear location model gave the best fit for Ghana. The quadratic location model gave the best fit for Benin, Cabo Verde, Cote d’Ivoire, Guinea, Mauritania and Nigeria. The linear location and linear scale model gave the best fit for Sierra Leone. The quadratic location and linear scale model gave the best fit for Mali. The quadratic location and quadratic scale model gave the best fit for Senegal.

**Figure 4:**
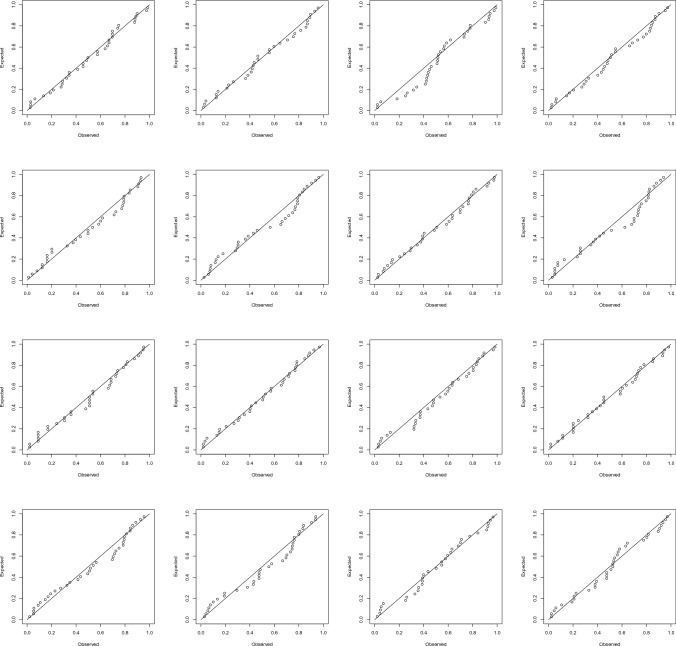
Probability plots for the best fitting models in Table 3 (first row, first column: Benin; first row, second column: Burkina Faso; first row, third column: Cabo Verde; first row, fourth column: Cote d’Ivoire; second row, first column: Gambia; second row, second column: Ghana; second row, third column: Guinea; second row, fourth column: Guinea-Bissau; third row, first column: Liberia; third row, second column: Mali; third row, third column: Mauritania; third row, fourth column: Niger; fourth row, first column: Nigeria; fourth row, second column: Senegal; fourth row, third column: Sierra Leone; fourth row, fourth column: Togo).

The probability plots of the best fitting models are shown in Fig. 4, contrast them with the plots in Fig. 3. The plots show that best fitting models are reasonable. The *p*-values of the Kolmogorov-Smirnov test^33,34^ were 0.055, 0.023, 0.084, 0.067, 0.075, 0.026, 0.072, 0.033, 0.077, 0.056, 0.046, 0.052, 0.065, 0.060, 0.098 and 0.073. The level of significance used was 0.05.

**Figure 5:**
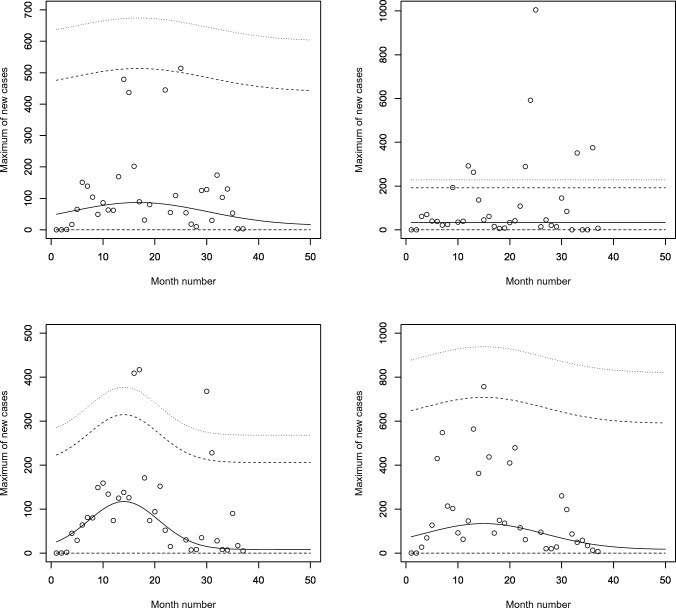
Fitted 2.5, 5, 50, 95 and 97.5 percent quantile functions versus month number (counting from January of 2020) for the best fitting models for Benin (top left), Burkina Faso (top right), Cabo Verde (bottom left) and Cote d’Ivoire (bottom right).

**Figure 6:**
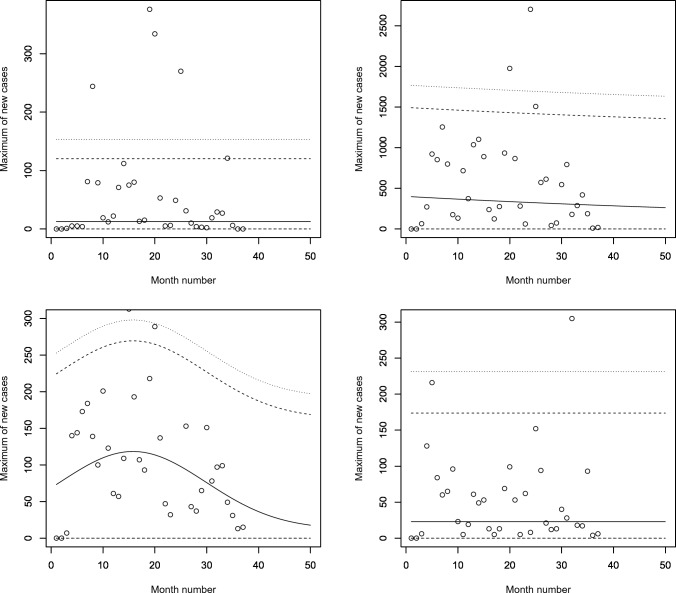
Fitted 2.5, 5, 50, 95 and 97.5 percent quantile functions versus month number (counting from January of 2020) for the best fitting models for Gambia (top left), Ghana (top right), Guinea (bottom left) and Guinea-Bissau (bottom right).

**Figure 7:**
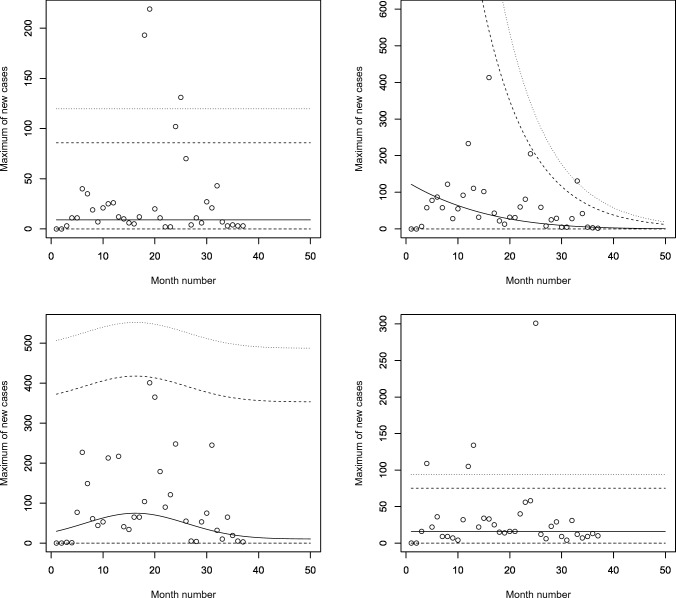
Fitted 2.5, 5, 50, 95 and 97.5 percent quantile functions versus month number (counting from January of 2020) for the best fitting models for Liberia (top left), Mali (top right), Mauritania (bottom left) and Niger (bottom right).

**Figure 8:**
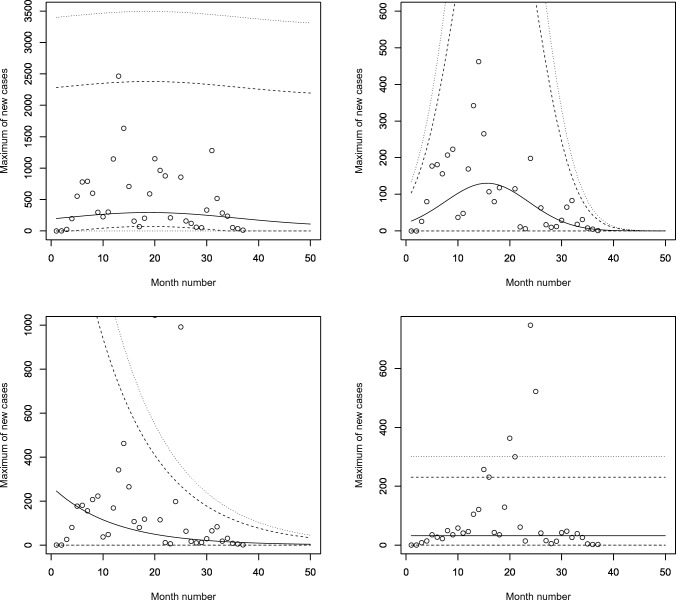
Fitted 2.5, 5, 50, 95 and 97.5 percent quantile functions versus month number (counting from January of 2020) for the best fitting models for Nigeria (top left), Senegal (top right), Sierra Leone (bottom left) and Togo (bottom right).

Figures 5, 6, 7 and 8 plot the 2.5, 5, 50, 95 and 97.5 percent quantile functions versus the month number ranging from 1 to 50 for the best fitting models. The *q*th percent quantile function for $q = 2.5, 5, 50, 95, 97.5$ was computed by

11 $$\begin{aligned} \displaystyle \widehat{Q} (q) = \left\{ \begin{array}{ll} \displaystyle 0, &{} {\text{if}}\, q \le \widehat{p}, \\ \\ \displaystyle \widehat{\mu } + \frac{\widehat{\sigma }}{\widehat{a_3}} \left\{ \left[ -\log \left( q - \widehat{p} \right) \right] ^{-\widehat{a_3}} - 1 \right\} , &{} {\text{if}}\, q > \widehat{p}, \end{array} \right. \end{aligned}$$

where $\widehat{\mu }$ is one of

$$\begin{aligned} \displaystyle \widehat{\mu } = \left\{ \begin{array}{ll} \displaystyle \exp \left( \widehat{a_1} \right) , &{} \text{ for } \text{ constant } \text{ model, } \\ \\ \exp \left[ \widehat{a_1} + \widehat{b_1} \cdot \text{ month } \text{ no } \right] , &{} \text{ for } \text{ linear } \text{ location } \text{ model, } \\ &{} \text{ linear } \text{ location } \text{ and } \text{ linear } \text{ scale } \text{ model, } \\ \\ \exp \left[ \widehat{a_1} + \widehat{b_1} \cdot \text{ month } \text{ no } + \widehat{c_1} \cdot {\text{ month } \text{ no }}^2 \right] , &{} \text{ for } \text{ quadratic } \text{ location } \text{ model, } \\ &{} \text{ quadratic } \text{ location } \text{ and } \text{ linear } \text{ scale } \text{ model, } \\ &{} \text{ quadratic } \text{ location } \text{ and } \text{ quadratic } \text{ scale } \text{ model } \end{array} \right. \end{aligned}$$

and $\widehat{\sigma }$ is one of

$$\begin{aligned} \displaystyle \widehat{\sigma } = \left\{ \begin{array}{ll} \displaystyle \exp \left( \widehat{a_2} \right) , &{} \text{ for } \text{ constant } \text{ model, } \\ &{} \text{ for } \text{ linear } \text{ location } \text{ model, } \\ &{} \text{ for } \text{ quadratic } \text{ location } \text{ model, } \\ \\ \exp \left[ \widehat{a_2} + \widehat{b_2} \cdot \text{ month } \text{ no } \right] , &{} \text{ linear } \text{ location } \text{ and } \text{ linear } \text{ scale } \text{ model, } \\ &{} \text{ quadratic } \text{ location } \text{ and } \text{ linear } \text{ scale } \text{ model, } \\ \\ \exp \left[ \widehat{a_2} + \widehat{b_2} \cdot \text{ month } \text{ no } + \widehat{c_2} \cdot {\text{ month } \text{ no }}^2 \right] , &{} \text{ quadratic } \text{ location } \text{ and } \text{ quadratic } \text{ scale } \text{ model. } \end{array} \right. \end{aligned}$$

The monthly maximum of new cases for Benin initially increases and then decreases. It is highest in April 2021. The monthly maximum of new cases for Cabo Verde also initially increases and then decreases. It is highest in January 2021. The monthly maximum of new cases for Cote d’Ivoire also initially increases and then decreases. It is highest in February 2021.

The monthly maximum of new cases for Ghana steadily decreases. The location parameter estimate decreases by a multiple of 0.348 for every unit increase in month number. The monthly maximum of new cases for Guinea initially increases and then decreases. It is highest in March 2021.

The monthly maximum of new cases for Mali steadily decreases. Both the location parameter estimate and scale parameter estimate steadily decrease. The latter decreases by a multiple of $1.528 \times 10^{-5}$ for every unit increase in month number. The monthly maximum of new cases for Mauritania initially increases and then decreases. It is highest in March 2021.

The monthly maximum of new cases for Nigeria initially increases and then decreases. It is highest in July 2021. The monthly maximum of new cases for Senegal initially increases and then decreases. The corresponding location parameter estimate reaches its highest value in February 2021. The corresponding scale parameter estimate reaches its highest value in April 2021. The corresponding quantile estimates appear to reach their highest values in March 2021. The monthly maximum of new cases for Sierra Leone steadily decreases. The location parameter estimate decreases by a multiple of $3.469 \times 10^{-5}$ for every unit increase in month number. The scale parameter estimate decreases by a multiple of $1.204 \times 10^{-4}$ for every unit increase in month number.

Table 5 gives the limit of (11) as the month number is taken to infinity. The 2.5 percent and 5 percent quantiles are equal to zero for all the ten countries. Quantiles of all levels are equal to zero for Mali, Senegal and Sierra Leone. Among the remaining countries, the 50th, 95th and 97.5th percent quantiles are smallest for Cabo Verde, followed by Mauritania, Benin, Cote d’Ivoire, Guinea, Ghana and Nigeria.

**Table 5:** Asymptotic limits of *q* percent quantile.

Country	$q = 2.5$	$q = 5$	$q = 50$	$q = 95$	$q = 97.5$
Benin	0	0	13	440	601
Cabo Verde	0	0	8	206	268
Cote d’Ivoire	0	0	16	590	821
Ghana	0	0	63	1158	1433
Guinea	0	0	16	590	821
Mali	0	0	0	0	0
Mauritania	0	0	10	353	487
Nigeria	0	0	68	2153	3270
Senegal	0	0	0	0	0
Sierra Leone	0	0	0	0	0

## Conclusions

We have modeled the monthly maximums of daily new cases from Benin, Burkina Faso, Cabo Verde, Cote d’Ivoire, Gambia, Ghana, Guinea, Guinea-Bissau, Liberia, Mali, Mauritania, Niger, Nigeria, Senegal, Sierra Leone and Togo using the generalized extreme value distribution. Two of the three parameters of this distribution were allowed to vary linearly or quadratically with respect to month number to account for trends. Ten of the countries (Ghana, Benin, Cabo Verde, Cote d’Ivoire, Guinea, Mauritania, Nigeria, Sierra Leone, Mali and Senegal) exhibited significant downward trends with respect to month number. Ghana exhibited a linear trend in its location parameter. Benin, Cabo Verde, Cote d’Ivoire, Guinea, Mauritania and Nigeria exhibited a quadratic trend in its location parameter. Sierra Leone exhibited a linear trend in its location and scale parameters. Mali exhibited a quadratic trend in its location parameter and a linear trend in its scale parameter. Senegal exhibited a quadratic trend in its location and scale parameters.

We have provided estimates of 2.5 percent, 5 percent, 50 percent, 95 percent and 97.5 percent quantiles for all the sixteen countries for up to March of 2024. We have also provided asymptotic limits of these quantiles as the month number approaches infinity. The goodness of the fitted models was examined by probability plots and the Kolmogorov-Smirnov test.

It is of concern that Burkina Faso, Gambia, Guinea-Bissau, Liberia, Niger and Togo did not exhibit significant downward trends in monthly maximums of daily new cases. It is also of concern that the asymptotic limits of 50 percent, 95 percent and 97.5 percent quantiles are strictly positive for Benin, Cabo Verde, Cote d’Ivoire, Ghana, Guinea, Mauritania and Nigeria. The governments of these countries may want to take appropriate actions to reduce new cases of COVID-19.

Future work are to: (i) extend the extreme value analysis of daily new cases to other countries in Africa; (ii) provide multivariate extreme value analysis of daily new cases from multiple countries; (iii) provide spatial and temporal extreme value analysis of daily new cases across the continent; (iv) extend the analysis for daily new deaths.

## Data Availability

The data used can be obtained from the corresponding author.

## Code availability

The code used can be obtained from the corresponding author.
